# 4kg solid replaces 80L liquid cleaner – innovative cleaner system for automated instrument reprocessing

**DOI:** 10.1186/1753-6561-5-S6-P40

**Published:** 2011-06-29

**Authors:** C Stingl, S Andermatt

**Affiliations:** 1 Ecolab, Wallisellen, Switzerland

## Introduction / objectives

SolidSafe System, an innovative cleaner system, has been developed for automated instrument reprocessing, consisting of cleaning and neutralisation products to be operated with a dispenser. Sustainability, easy and safe handling are core system benefits in addition to necessary application features a cleaning agent should have.

The environmental footprint is strongly influenced by offering water-free solid chemistry in 4 kg capsules. This solid chemistry is diluted to a liquid concentrate on site which is dosed from washer-disinfectors (WDs) for the reprocessing of medical instruments. The waste of empty plastic is reduced by 96%. This system offers a 28 fold reduction of energy for transportation as water based products no longer have to be shipped to the customer site.

## Methods

From an ergonomic perspective, chemicals in concentrated and solidified form are preferred to the current liquid system in the central sterile department (CSD) of hospitals as evidenced by a study by the German Insitute for Occupational Health and Saftey (IFA). With current liquid products supplied in 5 to 200L cans, the system minimizes change intervals as 1 solid capsule (4 kg) equals up to 80L of liquid concentrate.

## Results

Easily installed in different CSD set ups, the dispenser measures and monitors the concentration via conductivity device and ensures that the product is only released for use in the WDs when the defined concentration is reached. Data on the batch productions of liquid concentrate is saved in the dispenser system and can be down loaded as needed.

## Conclusion

The SolidSafe System is the first in Europe to offer a dispenser with high accuracy and reliability in combination with the solid and high concentrated cleaning products MetalClean and AlkalineClean and the neutralisation product NeutraPlus.

## Disclosure of interest

None declared.

